# Effect of Fecal Microbiota Transplantation Combined With Mediterranean Diet on Insulin Sensitivity in Subjects With Metabolic Syndrome

**DOI:** 10.3389/fmicb.2021.662159

**Published:** 2021-06-10

**Authors:** Annefleur M. Koopen, Eduardo L. Almeida, Ilias Attaye, Julia J. Witjes, Elena Rampanelli, Soumia Majait, Marleen Kemper, Johannes H. M. Levels, Alinda W. M. Schimmel, Hilde Herrema, Torsten P. M. Scheithauer, Werner Frei, Lars Dragsted, Bolette Hartmann, Jens J. Holst, Paul W. O’Toole, Albert K. Groen, Max Nieuwdorp

**Affiliations:** ^1^Department of Internal Medicine and (Experimental) Vascular Medicine, Amsterdam University Medical Center, Location Academic Medical Center, Amsterdam, Netherlands; ^2^APC Microbiome Ireland, School of Microbiology, University College Cork, Cork, Ireland; ^3^Department of Nutrition, Exercise and Sports, Faculty of Science, University of Copenhagen, Frederiksberg, Denmark; ^4^Novo Nordisk Foundation Center for Basic Metabolic Research, Department of Biomedical Sciences, University of Copenhagen, Copenhagen, Denmark; ^5^Department of Internal Medicine, Diabetes Center, Amsterdam University Medical Center, Location VU University Medical Center, Amsterdam, Netherlands

**Keywords:** gut microbiota, fecal microbiota transplantation, mediterranean diet, metabolic syndrome, insulin sensitivity

## Abstract

**Background:**

Recent studies demonstrate that a Mediterranean diet has beneficial metabolic effects in metabolic syndrome subjects. Since we have shown that fecal microbiota transplantation (FMT) from lean donors exerts beneficial effects on insulin sensitivity, in the present trial, we investigated the potential synergistic effects on insulin sensitivity of combining a Mediterranean diet with donor FMT in subjects with metabolic syndrome.

**Design:**

Twenty-four male subjects with metabolic syndrome were put on a Mediterranean diet and after a 2-week run-in phase, the subjects were randomized to either lean donor (*n* = 12) or autologous (*n* = 12) FMT. Changes in the gut microbiota composition and bacterial strain engraftment after the 2-week dietary regimens and 6 weeks post-FMT were the primary endpoints. The secondary objectives were changes in glucose fluxes (both hepatic and peripheral insulin sensitivity), postprandial plasma incretin (GLP-1) levels, subcutaneous adipose tissue inflammation, and plasma metabolites.

**Results:**

Consumption of the Mediterranean diet resulted in a reduction in body weight, HOMA-IR, and lipid levels. However, no large synergistic effects of combining the diet with lean donor FMT were seen on the gut microbiota diversity after 6 weeks. Although we did observe changes in specific bacterial species and plasma metabolites, no significant beneficial effects on glucose fluxes, postprandial incretins, or subcutaneous adipose tissue inflammation were detected.

**Conclusions:**

In this small pilot randomized controlled trial, no synergistic beneficial metabolic effects of combining a Mediterranean diet with lean donor FMT on glucose metabolism were achieved. However, we observed engraftment of specific bacterial species. Future trials are warranted to test the combination of other microbial interventions and diets in metabolic syndrome.

## Introduction

With the continuous rise in obesity prevalence (NCD Risk Factor Collaboration, 2016a), the number of obese adults with metabolic syndrome and type 2 diabetes mellitus (DM2) has quadrupled over the last decade (NCD Risk Factor Collaboration, 2016b). The most important driver of metabolic syndrome is excess intestinal uptake of energy. Our gut microbiota is one of the factors that play an important role in the regulation of energy harvest and storage, in which gut microbiota from obese subjects has been postulated to yield more energy from diet compared to gut microbiota from lean subjects (Bäckhed et al., 2004; Turnbaugh et al., 2006). Colonization of germ-free mice with microbiota from obese littermates or obese humans has been shown to cause an increase in body weight, demonstrating that this obese phenotype is transmissible *via* fecal microbiota transplantation (FMT) (Turnbaugh et al., 2006; Ridaura et al., 2013). In humans, several studies have demonstrated that the gut microbiota composition of healthy subjects differs from that of subjects with metabolic syndrome and DM2 (Ley et al., 2006; Turnbaugh et al., 2006; Qin et al., 2012; Karlsson et al., 2013; Castaner et al., 2018). Furthermore, we have previously shown that transplantation of lean donor fecal microbiota can transiently improve insulin sensitivity in obese subjects with metabolic syndrome (Vrieze et al., 2012; Kootte et al., 2017). In recent years, our group has also demonstrated that gut microbiota diversity at baseline is an important determinant of the engraftment of donor bacteria, which subsequently affects the magnitude of metabolic response after FMT (Li et al., 2016; Kootte et al., 2017).

The composition of the gut microbiota is governed by a complex interplay of many independent factors, such as exercise, concomitant medication use, aging, and diet (Dominguez-Bello et al., 2010; Yatsunenko et al., 2012; Clarke et al., 2014; Falony et al., 2016). Dietary composition and intake are considered the most important contributing factors to the altered diversity of intestinal microbes (De Filippo et al., 2010; Gentile and Weir, 2018). Indeed, prior data have clearly shown that the gut microbiota rapidly changes after alterations in diet (David et al., 2014; Zeevi et al., 2015), with an increase in gut microbiota diversity upon a healthy (high-fiber) diet (Cotillard et al., 2013). A Mediterranean diet is such a high-fiber diet that has been associated with many beneficial health effects, such as reduced cardiovascular (Estruch et al., 2018) and DM2 risk (Salas-Salvadó et al., 2011; Haro et al., 2016). Also, beneficial changes in the microbiota composition after a Mediterranean diet have been reported (De Filippis et al., 2015; Haro et al., 2016; Ghosh et al., 2020). A recent randomized controlled trial has investigated the effects of an 8-week adherence to a Mediterranean diet in 82 obese subjects (Meslier et al., 2020). In line with earlier studies, a beneficial effect on the plasma cholesterol levels was observed as well as a number of changes in fecal microbiota, while the effect on insulin sensitivity was marginal. Although many studies have demonstrated that the type of diet has a great impact on the gut microbiota composition, none of these previous intervention studies combine modulation of dietary intake with reestablishment of a healthy microbiome by means of lean donor FMT.

In the present study, we therefore hypothesized that a Mediterranean diet may help beneficial microbes to better engraft, enabling long-term and greater effects after FMT. We thus performed lean healthy donor FMT preceded by a controlled Mediterranean diet and studied possible synergistic effects on the gut microbiota composition in subjects with metabolic syndrome. Secondary objectives were intervention-driven changes in both hepatic and peripheral insulin sensitivity, postprandial plasma concentrations of the incretin glucagon-like peptide-1 (GLP-1), subcutaneous adipose tissue (AT) inflammation, and plasma metabolites.

## Materials and Methods

### Study Population

We recruited obese treatment-naive Caucasian male subjects (age, 21–65 years) with a body mass index (BMI) between 30 and 43 kg/m^2^ with otherwise normal health *via* local newspaper advertisements. All subjects had to meet the inclusion criteria of metabolic syndrome (Alberti et al., 2006); requiring to meet at least three out of the following five criteria: fasting plasma glucose ≥ 5.6 mmol/L, triglycerides ≥ 1.7 mmol/L, waist circumference ≥ 102 cm, high-density lipoprotein (HDL) cholesterol ≤ 1.04 mmol/L, and/or blood pressure ≥ 130/85 mmHg. The main exclusion criteria were the use of any type of medication, smoking, alcohol abuse, a history of cardiovascular event or cholecystectomy, and being unmotivated or unable to adhere to the diet. Moreover, eligible FMT donors were healthy Caucasian men (age, 18–65 years) with a BMI between 18.5 and 25 kg/m^2^. Donors were thoroughly screened as previously described (Kootte et al., 2017) for the presence of infectious diseases in blood and feces and completed questionnaires regarding medical, sexual, family and travel history, and bowel habits, as previously described (Cammarota et al., 2017). Written informed consent was obtained from all subjects. The study was approved by the local Institutional Review Board of the Amsterdam University Medical Center (Amsterdam UMC) in Amsterdam, the Netherlands, and conducted at the AMC in accordance with the Declaration of Helsinki. The study was registered at the Dutch Trial Register (NTR 5983).

### Study Design

**FIGURE 1:**
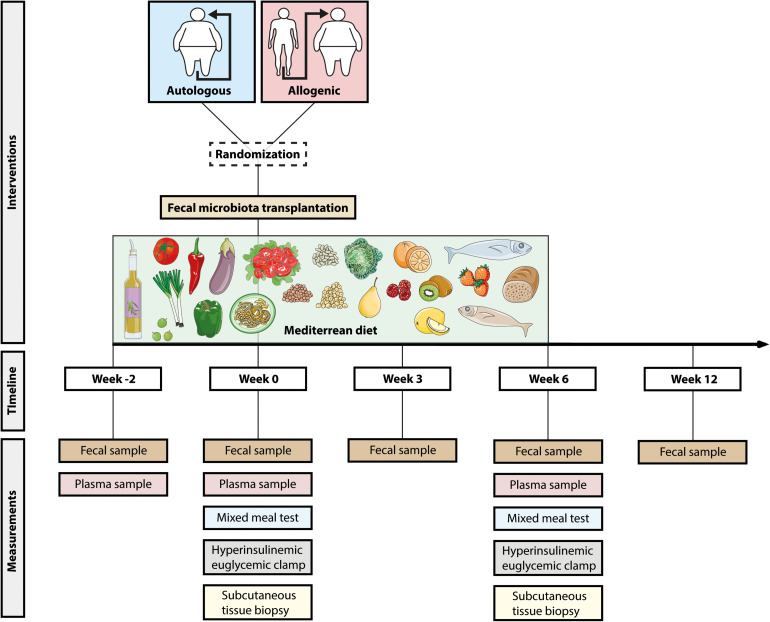
Study design. Overview of the study. All subjects adhered to the Mediterranean diet for 8 weeks (from W–2 to W6). After 2 weeks (W0), the subjects were randomized to autologous or allogeneic fecal microbiota transplantation. Before the start of the diet (W–2), the first fecal and blood samples were collected. At W0 (before fecal microbiota transplantation, FMT) and W6, we performed a mixed meal test, hyperinsulinemic euglycemic clamp, and a subcutaneous tissue biopsy.

The design of the study is presented in Figure 1. We conducted a double-blind randomized controlled single-center trial. All male metabolic syndrome subjects adhered to a Mediterranean diet for a total of 8 weeks with a 2-week run-in period (week −2 to week 0). After these 2 weeks, the subjects were randomized (using computerized randomization) to either an allogeneic FMT (receiving the feces of a lean healthy donor) or autologous FMT (receiving their own feces). At weeks −2, 0, and 6 we collected anthropomorphic data and blood and fecal samples. Three weeks after the FMT, we collected an additional fecal sample, and at week 12 (6 weeks after cessation of the diet), the subjects visited our center for the last time to provide a final fecal sample. In week 0, we performed tests to determine postprandial lipid metabolism, subcutaneous adipose tissue inflammation, and insulin sensitivity, which we repeated at week 6. The sample size of *n* = 12 was based on data from our previous FMT clinical trials in which absolute improvements in insulin sensitivity from 26.2 to 45.3 μmol L^–1^ min^–1^ (Vrieze et al., 2012) and from 25.8 to 28.8 μmol L^–1^ min^–1^ (Kootte et al., 2017) were observed upon lean donor FMT.

### Week −2: Start of Mediterranean Diet

The Mediterranean diet guidelines used in this study were based on the PREDIMED Study (Estruch et al., 2018), although adapted slightly to Dutch settings in consultation with the clinical dietitian who was part of the research team. The diet contained the following components: two or more daily servings of vegetables, two or more daily servings of fresh fruits, three or more weekly servings of legumes, two or more weekly servings of fish or seafood, abundant use of olive oil for cooking and dressing dishes, and daily consumption of nuts. Negative recommendations were given about red meat, cream, butter, carbonated and/or sugared beverages, and pastries and industrial bakery products (see Supplementary File 1). To enhance adherence to the Mediterranean diet, the subjects were provided food boxes developed in collaboration with a Dutch food box company (de Krat, Amsterdam, Netherlands). The boxes were suited for two adults so that meals for partners did not have to be prepared separately, thereby facilitating compliance. The food boxes contained all the ingredients for five main meals, five times lunch, and extra supplementation of fruit, extra virgin olive oil (one bottle of 500 ml per week), and raw unsalted mixed nuts (500 g per week). The recipes and contents of the boxes met our Mediterranean guidelines and were all checked by the clinical dietitian. For the remaining 2 days of the week, subjects could prepare their own food, provided that they adhered to the Mediterranean guidelines provided by the dietitian. The same held true for breakfast and any snacks.

All subjects visited the Amsterdam UMC clinical dietitian before they started the Mediterranean diet. During this intake, the subjects were counseled on the Mediterranean diet plus food boxes and received personal advice based on the online nutritional diary^1^ they completed beforehand. During the 2-week run-in period that followed, the decision was made whether a subject could continue with the study based on motivation and adherence. During the diet phase, the subjects were asked to fill out the online food diary at three different time points for at least 2 days (which were checked by the dietitian) and had telephone calls with the dietitian for at least two occasions and more if necessary. The 3 days before the last visit, the subjects completed the nutritional diary for a final time. From the online nutritional diary, the total energy, carbohydrate, protein, fiber, total fat, and saturated fat intakes were calculated for the pre-diet, during, and post-diet stages.

### Mixed Meal Test

Mixed meal test (MMT) was performed at week 0 (study day 1) and repeated at week 6. After an overnight fast, baseline blood samples were drawn from an inserted intravenous catheter in a distal arm vein. Hereafter, the subjects immediately ingested a standardized liquid high-fat meal containing 626 kcal (61% fat, 33% carbohydrates, and 6% proteins) within 5 min, as previously described (Reijnders et al., 2016; Kootte et al., 2017). For the next 4 h, blood samples were drawn every 30 min for postprandial metabolism and were stored at −80°C. Before the start of the MMT, abdominal subcutaneous adipose tissue was aspirated as previously described (De Groot et al., 2019). All subjects provided a fecal sample for gut microbiota and short-chain fatty acid (SCFA) analysis on this day or study day 2.

### Two-Step Hyperinsulinemic Euglycemic Clamp

On the second study day of week 0 and week 6, a two-step hyperinsulinemic euglycemic clamp test with stable isotopes was performed to measure hepatic and peripheral insulin sensitivity, as described previously (Vrieze et al., 2012; Kootte et al., 2017). After an overnight fast, intravenous catheters were inserted into a distal vein of both arms. One catheter was used for arterialized venous blood withdrawal using a heated-hand box (60°C); the other catheter was used for infusion of the glucose tracer, glucose, and insulin. Prior to infusion, baseline blood samples were drawn to determine background isotope enrichment. Hereafter, a continuous infusion of [6,6-^2^H_2_] glucose (prime, 11 μmol kg^–1^; continuous, 0.11 μmol kg^–1^ min^–1^) was started and continued until the end of the experiment. After blood withdrawal at 2 h of equilibration, infusion of insulin (Actrapid; Novo Nordisk Farma B.V., Alphen aan den Rijn, Netherlands) at a rate of 20 mU m^–2^ min^–1^ was started. Plasma glucose concentrations were measured every 10 min and infusion of a 20% glucose solution enriched with 1% [6,6-^2^H_2_] glucose was started to maintain a plasma glucose concentration of 5 mmol/L. After 2 h of insulin infusion, five repetitive blood samples were drawn with an interval of 5 min to determine glucose enrichments, glucoregulatory hormones, and free fatty acids. Hereafter, the insulin infusion rate was increased to 60 mU m^–2^ min^–1^ and continued for another 2 h, after which five repetitive blood samples were drawn again. Plasma samples were stored at −80°C for later analyses. Resting energy expenditure (REE) was measured using indirect calorimetry during the basal and hyperinsulinemic state. Oxygen and carbon dioxide productions were measured for 20 min using a ventilated hood system (Vmax Encore 29; SensorMedics, Anaheim, CA, United States).

### Gene Expression of Subcutaneous Tissue Biopsy

At both week 0 and week 6, abdominal subcutaneous adipose tissue was aspirated using a hollow needle and a 50-ml syringe. TriPure Isolation Reagent was used to isolate RNA according to the protocol of the manufacturer (Roche, Mannheim, Germany). SensiFAST cDNA Synthesis Kit (Bioline, London, United Kingdom) was used to prepare complementary DNA (cDNA) and SensiFAST SYBR No-ROX Kit (Bioline, London, United Kingdom) was used to measure mRNA expression. The expression levels were normalized to RPLP0 (ribosomal protein lateral stalk, subunit P0). The primers for *IL-10*, *CCL2*, *CD68*, *CD11c*, *IRS1*, *TNF*, and *IL-6* are presented in Supplementary Table 1.

### Fecal Microbiota Transplantation

Fecal microbiota transplantation was performed on the third study day of week 0. Gastroduodenoscopy and the positioning of the nasoduodenal tube and FMT on the third day of the first week were all performed as described previously (Kootte et al., 2017). *Via* gastroduodenoscopy, a nasoduodenal tube was placed and its position was checked by an abdominal X-ray. Hereafter, bowel lavage was started with infusion of (usually 2–3 L) dissolved macrogol/electrolytes (Klean-Prep) through the nasoduodenal tube to clean the intestines of fecal material. At this point, the subject was randomized in a double-blinded fashion to receive either the feces from the assigned lean donor (allogeneic FMT) or his own feces (autologous FMT), both delivered as fresh fecal sample the same morning. An independent colleague with access to the randomization list made sure that the researcher used the (blinded) container with the assigned feces. The feces was mixed with a saline solution (0.9% NaCl) until fully homogenized and sieved to remove all debris. The obtained homogenous solution was subsequently stored in a sterile 500-ml bottle. After complete bowel lavage, 500 ml of the dissolved fecal content was infused *via* the nasoduodenal tube. All procedures (MMT, hyperinsulinemic clamp test, and subcutaneous tissue biopsy) were performed at both week 0 and week 6, with the exception of the FMT.

### Gut Microbiota Sequencing Analysis

#### DNA Extraction

Genomic DNA was extracted from 0.25 g of fecal samples using the repeat bead beating (RBB) method of Yu and Morrison^2^, with the following modifications. Three types of sterile zirconia beads (Thistle Scientific, Glasgow, United Kingdom) were used (0.5 g in total; one 3.0-mm bead, 0.1 g of 0.5-mm beads, and 0.3 g of 0.1-mm beads). Fecal samples were homogenized three times for 60 s at maximum speed on a Mini-Beadbeater-24^TM^ (Thistle Scientific, Glasgow, United Kingdom), with the samples cooled on ice for 60 s in between bead-beating cycles. The supernatants of two bead-beating rounds were pooled and incubated with 350 μl of 7.5 M ammonium acetate (Sigma, St. Louis, MO, United States) on ice. The extraction proceeded as per the RBB protocol using Qiagen’s DNeasy^®^ Blood & Tissue Kit (Qiagen, West Sussex, United Kingdom) according to the manufacturer’s instructions for the final DNA purification (without the lysis steps and eluted in 100 μl of AE buffer).

#### DNA Library Preparation

Genomic DNA was quantified using the Qubit dsDNA high-sensitivity assay kit (Invitrogen – Carlsbad, California, United States). Samples were prepared for shotgun metagenomic sequencing using the Illumina Nextera XT library preparation kit and following the manufacturer’s instructions. Unique Nextera XT 8-nt dual indices were used for multiplexing (Illumina, San Diego, CA, United States). Libraries were pooled to an equimolar concentration and sequenced by Edinburgh Genomics (Edinburgh, United Kingdom) using a 2 × 150-bp paired-end method on an Illumina NovaSeq 6000 platform and aiming to achieve ∼4–5 Gbp of sequencing data per sample.

The raw reads were initially processed using the KneadData tool (version 0.7.2^3^) for read quality trimming, filtering, and also the removal of potential contaminant reads with the recommended settings. Microbial community composition profiling was performed using the MetaPhlAn 2.0 pipeline (Truong et al., 2015), while the HUMAnN 2.0 pipeline (Franzosa et al., 2018) was employed for functional profiling and, more specifically, for gene count determination. The resulting data from these pipelines were processed using R (version 4.0.2) (R Core Team, 2020) in the RStudio IDE (version 1.3.1093) (R Studio Team, 2020). Alpha diversity indices were computed using the *phyloseq* (version 1.34.0) (McMurdie and Holmes, 2013) and *vegan* (version 2.5–6) (Oksanen et al., 2017) packages, and beta diversity was computed using Spearman’s distances between samples.

### Fecal Short-Chain Fatty Acid and Bile Acid Measurements

Fecal SCFA levels were measured using high-performance liquid chromatography (HPLC) with UV detection according to the method of De Baere (De Baere et al., 2013). In addition, for all samples, the dry weights were determined after freeze drying a homogenized fecal aliquot for 24 h. The SCFA measurements were corrected for the difference in the wet and dry weights for each sample. We also measured the concentrations of seven bile acids and the neutral sterols cholesterol, dihydrocholesterol, and coprostanol in the stool samples, as previously described (Jakulj et al., 2016).

### Plasma Metabolites and GLP1 Levels

Untargeted metabolomics profiling was performed by Metabolon (Durham, NC, United States) using ultra high-performance liquid chromatography coupled to tandem mass spectrometry (UPLC-MS/MS), as previously described (Koh et al., 2018). This resulted in 756 annotated plasma metabolites. Raw data were normalized and subsequently rescaled to set the median equal to 1. Missing values, mainly caused by measurements below the detection limits, were imputed. With regard to plasma GLP-1, the concentrations were determined in postprandial samples in the first 2 h of the mixed meal test (at 0, 30, and 120 min). The levels of total GLP-1 were measured by Holst group with ELISA (cat no. 10-1278-01; Mercodia, Sweden). All quality controls provided by the manufacturer were within the allowed limits. All samples from the same individual were measured in the same assay run.

### Statistical Analysis

The clinical parameters measured were not normally distributed and are thus presented as medians and interquartile ranges. Non-parametric tests were used for statistical testing: Wilcoxon signed-rank test was used for within-group comparisons and the Mann–Whitney *U* test was used for between-group comparisons. Postprandial results are described as incremental area under the curves (iAUC). Statistical analyses were performed using SPSS Statistics software, version 25. *P*-values < 0.05 were considered statistically significant.

Statistical analyses of the microbiome and metabolome data were performed in R (R Core Team, 2020) using the RStudio IDE (R Studio Team, 2020). Statistically significant differences between multiple groups were computed employing the non-parametric Kruskal–Wallis’ test, with pairwise comparisons performed using the Wilcoxon’s test, using the *ggpubr* package (version 0.4.0) (Kassambara, 2020). Multiple hypothesis testing *p*-values were adjusted for false discovery rate using the Benjamini and Hochberg method, as indicated. Principal coordinates analysis plots were computed using the *ade4* package (version 1.7–16) (Thioulouse et al., 2018), and statistically significant differences between groups were determined employing permutational multivariate analysis of variance (MANOVA) *via* the *adonis* function from the *vegan* package (Oksanen et al., 2017). Unless indicated otherwise, *p*-values < 0.05 were considered statistically significant. Plots were generated in R using the *ggplot2* (version 3.3.2) (Wickham, 2016), *ggpubr* (Kassambara, 2020), and *ComplexHeatmap* (version 2.6.0) (Gu et al., 2016) packages.

## Results

We included a total of 28 male metabolic syndrome subjects from November 2016 until September 2018, of whom four were excluded due to technical difficulties with clinical measurements (two subjects), non-compliance to the Mediterranean diet (one subject), and withdrawal because of personal reasons before the start of the diet (one subject). The baseline characteristics of the 24 randomized patients are presented in Table 1. There were no significant differences in baseline characteristics between subjects who later received an autologous or allogeneic FMT (Table 1). Moreover, we included five healthy subjects who served as FMT donors in multiple metabolic syndrome subjects, with eventually a range of one to four recipients of allogeneic FMT per single donor.

**TABLE 1:** Baseline characteristics at week−2 for the study subjects and separated by fecal microbiota transplantation (FMT) group.

	**Total group (*n* = 24)**	**Autologous (*n* = 12)**	**Allogeneic (*n* = 12)**
Male gender (%)	100	100	100
Age (years)	51.5 (47–58.8)	52.5 (47.3–54.8)	50.5 (46.3–60.0)
Weight (kg)	118.7 (104.2–129.3)	119.6 (108.9–129.3)	116.2 (100.2–129.4)
BMI (kg/m^2^)	34.0 (31.8–37.3)	35.4 (32.7–40.0)	33.06 (30.8–37.2)
Waist circumference (cm)	120 (113–131)	121.5 (112.3–129.5)	118 (111.8–132.5)
Blood pressure: systolic (mmHg)	150 (138–89)	152 (144–172)	147 (132–156)
Blood pressure: diastolic (mmHg)	93 (89–105)	96 (91–110)	91 (84–102)
Fasting glucose (mmol/L)	5.8 (5.5–6.4)	5.90 (5.53–6.36)	5.75 (5.53–6.53)
Insulin (pmol/L)	102 (62–124)	104 (67.2–120.8)	91 (53.1–192.2)
HOMA-IR	3.7 (2.3–4.6)	3.65 (2.63–4.48)	3.55 (2.20–7.23)
HbA1c (mmol/mol)	40 (36–41)	39.5 (37–40)	40 (36–41)
Cholesterol: total (mmol/L)	5.7 (4.6–6.3)	5.97 (4.80–6.36)	5.16 (4.59–6.24)
Cholesterol: HDL (mmol/L)	1.3 (1.1–1.5)	1.28 (0.97–1.52)	1.32 (1.10–1.45)
Cholesterol: LDL (mmol/L)	3.6 (2.9–4.2)	4.08 (2.69–4.48)	3.12 (2.92–4.12)
Cholesterol: triglycerides (mmol/L)	1.3 (1.0–1.8)	1.35 (1.15–2.12)	1.32 (1.01–1.77)

*Data are expressed as medians and interquartile ranges. There were no statistically significant differences in the baseline characteristic between subjects who later received an autologous or allogeneic FMT.*

### Adherence to Mediterranean Diet and Effects on Gut Microbiota Composition

**FIGURE 2:**
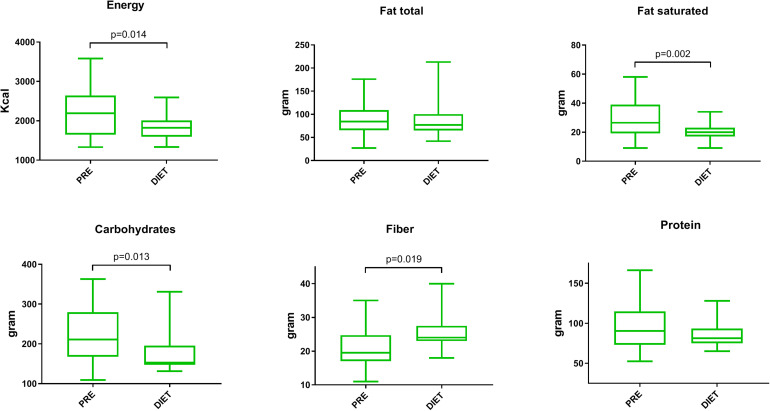
Changes in macronutrient intake following the Mediterranean diet. Changes in the daily macronutrient intake comparing the habitual diet (*PRE*) to the Mediterranean diet (*DIET*). For comparison, a paired Wilcoxon signed-rank test was used. Only statistically significant *p*-values are shown. *Box* and *whisker*, min–max.

Macronutrient intake before, during, and after the Mediterranean diet period were extracted from the self-reported online nutritional diaries (Figure 2 and Supplementary Table 2). The total energy intake of the whole group during the first 2 weeks on Mediterranean diet was significantly reduced compared to that of the habitual diet [from 2,193 (1,734–2,644) to 1,820 kcal (1,602–2,009) daily, *p* = 0.01]. During the diet phase, the subjects consumed significantly less saturated fat [from 28 (19–39) to 20 g (17–23), *p* = 0.01], with an unchanged total fat intake [from 85 (68–109) to 81 g (66–100), *p* = 0.67]. The subjects also significantly reduced their total amount of carbohydrates [from 216 (167–272) to 158 g (148–191), *p* = 0.01], but increased their fiber intake [from 20 (17–25) to 24 g (23–28), *p* = 0.01]. The decrease in protein intake was not significant [from 90 (73–115) to 81 g (75–94), *p* = 0.12] (Supplementary Table 2). When the subjects who were later randomized to the allogeneic FMT were compared with those in the autologous FMT group, no differences in the Mediterranean diet-induced changes in energy, fat, saturated fat, carbohydrate, and fiber intakes were observed.

**FIGURE 3:**
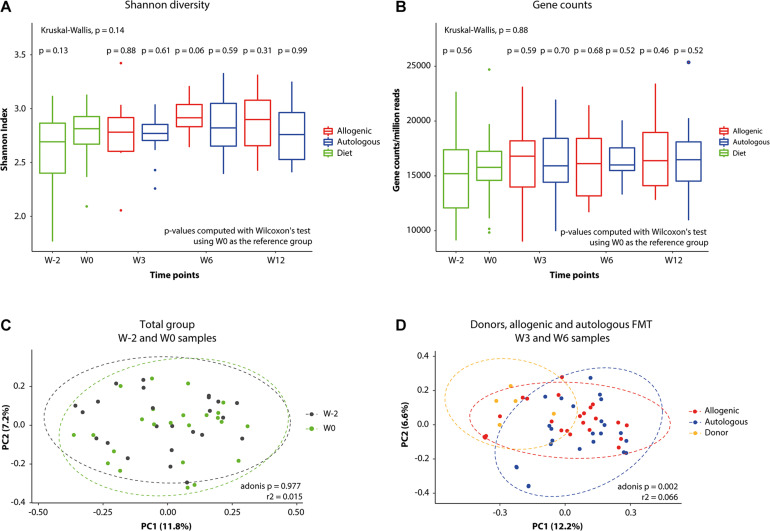
Changes in gut microbiota global measures. Differences in fecal microbiota alpha diversity. **(A)** Shannon index values. **(B)** Normalized gene counts of bacterial species. **(C,D)** Principal coordinates analysis (PCoA) plots graphically representing the microbiota compositional changes after **(C)** Mediterranean diet (from W–2 to W0) and after **(D)** fecal microbiota transplantation (FMT) on top of the Mediterranean diet.

With regard to any effect on the gut microbiota composition due to the Mediterranean diet, the values for alpha diversity (Shannon index) and gene counts are presented in Figures 3A,B. No effect of the Mediterranean diet on microbiota Shannon diversity or metagenome gene count was detectable over the 2-week period from the start of the Mediterranean diet for the whole group (W−2 to W0, *p* = 0.12 and *p* = 0.56, respectively). Furthermore, no clear shift in the overall microbiome relatedness (beta diversity) was observed upon the Mediterranean diet (W−2 to W0; Figure 3C). Following FMT, however, we did observe a clustering according to the source of FMT (Figure 3D).

**FIGURE 4:**
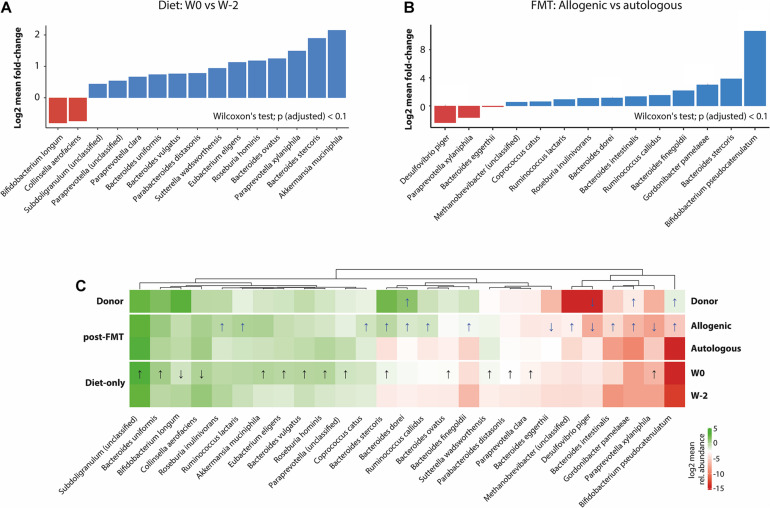
Alterations in the gut microbiota associated with treatments. **(A)** Significant changes (*p*_adjusted_ < 0.1) in the species relative abundance after the Mediterranean diet (W0 vs. W2). **(B)** Differences (*p*_adjusted_ < 0.1) between the allogeneic and autologous fecal microbiota transplantation (FMT) groups. **(C)** Heat map representing the log2-transformed 10% trimmed means of the bacterial species relative abundances in the different groups (donor, allogeneic FMT, autologous FMT, W0, and W–2). Only showing species with a statistically significant differential abundance between at least two groups (Wilcoxon’s test of significance with a *p*_adjusted_ < 0.1), after filtering out rare taxa (at least 0.01% abundance in at least 50% of the samples within each group). Statistically significant differential abundance values between W0 vs. W–2 (diet effect) are indicated with *black arrows*, while those between donor and allogeneic vs. autologous (W3, W6, and W12) (FMT effect) are indicated by *blue arrows*. Directionality of differential abundance (greater or less) is indicated either with an *arrow pointing up* or *arrow pointing down*, respectively. Interestingly, when comparing the donor and the allogeneic groups with the autologous group, some overlap is observed, including the direction of differential abundance, strongly indicating that these species could have been acquired from the donor group or are being antagonized by species acquired from the donor group.

Although we did not observe great changes in the overall microbial species richness and composition after 2 weeks of Mediterranean diet, subsequent gut microbiota analysis demonstrated marked changes in fecal abundance of several species 2 weeks after the start of the Mediterranean diet (Figure 4A). Adherence to the Mediterranean diet resulted in an increased abundance of several species, including *Bacteroides* species, *Akkermansia muciniphila*, and SCFA butyrate-producing *Roseburia hominis*, as well as a reduced fecal abundance of *Collinsella aerofaciens*.

Following allogeneic FMT (from W0 to W6), we observed in the recipient fecal microbiota an increase in several bacterial species, which were also present in the healthy donor microbiota (Figure 4C). Moreover, a significant differential enrichment in the gut microbial species was detected between the group receiving autologous FMT or allogeneic donor FMT, with the donor-derived microbiota being enriched in *Bifidobacterium pseudocatenulatum*, *Gordonibacter pamelaeae*, and *Bacteroides dorei* and deprived in *Desulfovibrio piger* with respect to autologous microbiota (Figures 4B,C).

### Effect of Combining FMT With Mediterranean Diet on Metabolic Parameters

**FIGURE 5:**
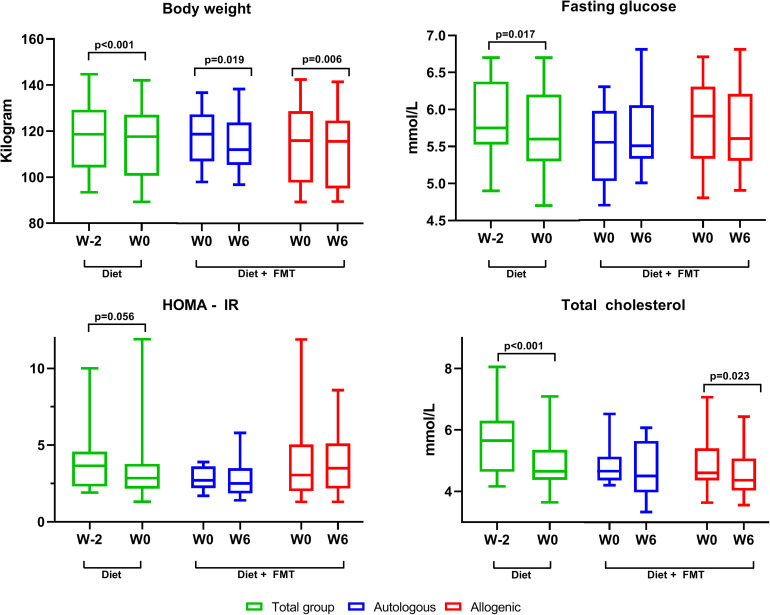
Changes in the clinical parameters. This figure shows changes in a subset of the clinically relevant parameters. In *green* are the changes in the first 2 weeks after initiation of the Mediterranean diet (for the whole group, since the subjects were not yet randomized). In *blue* and *red* are the changes after autologous or allogeneic fecal microbiota transplantation (FMT), respectively. *Box* and *whisker*, min–max. Only statistically significant *p*-values are shown. There were no significant differences between the autologous and allogeneic groups.

Changes in the metabolic parameters caused by the Mediterranean diet alone (from W−2 to W0) and, subsequently, the addition of the FMT (from W0 to W6) are presented in Table 2, with a subset of markers visualized in Figure 5. In the first 2 weeks of the diet, before the FMT, the body weight of the subjects significantly decreased [118.7 kg (104.2–129.3) at baseline vs. 117.7 kg (100.6–127.1) after a 2-week diet, *p* < 0.001]. Likewise, we found a significant reduction in the fasting blood glucose level [from 5.8 (5.5–6.4) to 5.6 mmol/L (5.3–6.2), *p* = 0.02], a numeric, but non-significant, decrease in the fasting insulin levels [from 102 (62–124) to 83 (57–107), *p* = 0.25], and a borderline significant decrease in homeostatic model assessment of insulin resistance (HOMA-IR) [from 3.7 (2.3–4.6) to 2.8 (2.2–3.8), *p* = 0.056]. Moreover, total cholesterol, high-density lipoprotein (HDL) cholesterol, and low-density lipoprotein (LDL) cholesterol were all significantly reduced after 2 weeks of Mediterranean diet intake. Similarly, dietary intervention diminished the total leukocyte counts and the systemic liver injury markers gamma-glutamyl transferase (GGT) and alkaline phosphatase (AP). After randomization to either an autologous or allogeneic FMT (from W0 to W6), both groups continued losing a significant amount of weight. The glucose levels remained stable, as well as the insulin levels and HOMA-IR. There was a small but statistically significant reduction in glycated hemoglobin HbA1c in the 6 weeks after both allogeneic and autologous FMT (8 weeks after starting the diet; Table 2).

**TABLE 2:** Clinical parameters expressed as medians and interquartile ranges.

	**Diet only**			**Diet + autologous FMT**			**Diet + allogeneic FMT**		
	**W−2 (*n* = 24)**	**W0 (*n* = 24)**	***p***	**W0 (*n* = 12)**	**W6 (*n* = 12)**	***p***	**W0 (*n* = 12)**	**W6 (*n* = 12)**	***p***
Weight (kg)	118.7 (104.2–129.3)	117.7 (100.6–127.1)	0.01	118.6 (106.9–127.1)	111.9 (105.3–123.7)	0.01	115.8 (97.6–128.5)	114.5 (95.1–124.4)	**0.01**
Fasting glucose (mmol/L)	5.8 (5.5–6.4)	5.6 (5.3–6.2)	**0.02**	5.6 (5.0–6.0)	5.5 (5.3–6.1)	0.62	5.9 (5.3–6.3)	5.6 (5.3–6.2)	0.40
Insulin (pmol/L)	102 (62–124)	83 (57–107)	0.2	81 (61–97)	69 (61–98)	0.46	86 (50–140)	100 (57–149)	0.72
HOMA-IR	3.7 (2.3–4.6)	2.8 (2.2–3.8)	**0.05**	2.7 (2.2–3.6)	2.5 (1.9–3.5)	0.64	3.0 (2.0–5.0)	3.5 (2.2–5.1)	0.39
HbA1c (mmol/mol)	40 (36–41)	39 (37–40)	0.2	38 (36–39)	36 (34–38)	**0.01**	40 (38–42)	37 (36–40)	**0.01**
Cholesterol: total (mmol/L)	5.7 (4.6–6.3)	4.7 (4.4–5.4)	**0.01**	4.7 (4.4–5.2)	4.5 (4.0–5.7)	0.2	4.6 (4.4–5.4)	4.4 (4.0–5.1)	**0.02**
Cholesterol: HDL (mmol/L)	1.3 (1.1–1.5)	1.1 (1.0–1.2)	**0.01**	1.1 (0.9–1.4)	1.2 (0.9–1.4)	0.50	1.1 (1.0–1.2)	1.1 (0.9–1.3)	0.53
Cholesterol: LDL (mmol/L)	3.6 (2.9–4.2)	3.0 (2.5–3.6)	**0.01**	3.1 (2.5–3.5)	3.1 (2.2–3.8)	0.43	3.0 (2.4–3.6)	2.9 (2.4–3.4)	0.06
Cholesterol: triglycerides (mmol/L)	1.3 (1.0–1.8)	1.2 (1.0–1.6)	0.5	1.3 (1.0–1.6)	0.9 (0.8–1.5)	0.07	1.2 (1.0–1.6)	1.1 (0.8–1.3)	0.31
CRP (mg/L)	2.2 (1.2–5.3)	2.6 (1.6–4.0)	0.96	2.6 (1.6–3.8)	2.5 (1.9–3.9)	0.66	2.7 (1.7–5.9)	3.5 (1.1–6.0)	0.37
Leukocytes (10^9^/L)	6.8 (5.5–7.6)	6.3 (4.7–7.3)	**0.01**	6.2 (4.9–7.2)	5.5 (4.7–6.3)	0.25	6.5 (4.2–7.9)	6.7 (4.7–8.7)	0.28
AP (U/L)	75 (68–96)	74 (63–84)	**0.01**	74 (65–91)	74 (63–88)	0.06	73 (58–78)	73 (62–84)	0.15
y-GT (U/L)	38 (34–61)	37 (27–55)	**0.01**	30 (22–41)	27 (19–42)	0.24	49 (29–75)	44 (25–60)	0.08
ASAT (U/L)	25 (21–28)	26 (24–31)	0.16	26 (24–31)	26 (22–31)	0.86	27 (23–32)	25 (23–26)	**0.03**
ALAT (U/L)	32 (22–39)	33 (27–43)	0.28	33 (27–26)	30 (23–33)	**0.02**	37 (23–46)	32 (20–44)	**0.02**

*Effect of Mediterranean diet only (W−2 vs. W0, whole group) and with the addition of fecal microbiota transplantation (FMT) (W0 vs. W6, split into autologous FMT and allogeneic FMT). There were no significant differences in any of the parameters between the autologous and allogeneic FMT groups. Significant differences (p < 0.05) emphazised in bold.*

Lastly, as low-grade systemic inflammation is an important pathologic feature of insulin resistance and adiposity, we examined the impact of FMT on inflammation at the systemic (plasma) and local (subcutaneous adipose tissue biopsies) levels. Independently of the source, FMT did not lead to alterations in circulating C-reactive protein (CRP) and leukocyte count (Table 2), nor did it affect the expressions of the inflammatory genes (*IL6*, *IL10*, *CD68*, *CCL2*, *IRS1*, *CD11c*, and *TNFA*) within subcutaneous AT (Supplementary Table 1 and Supplementary Figure 1).

#### Effect of FMT on Insulin Sensitivity and Postprandial Metabolism

Next, we evaluated the impact of FMT interventions on peripheral and hepatic insulin sensitivity. Peripheral insulin sensitivity, assessed as the insulin-induced glucose disposal rate (*R*_d_), was unaltered after either autologous or donor FMT (Supplementary Figure 2A): autologous *R*_d_ from 39.7 (33.2–53.1) to 44.4 μmol kg^–1^ min^–1^ (32.4–56.2, *p* = 0.88); allogeneic *R*_d_ from 41.0 (24.0–48.3) to 41.7 μmol kg^–1^ min^–1^ (34.8–49.5, *p* = 0.48). Similarly, hepatic insulin sensitivity measured as insulin-mediated suppression of endogenous glucose production (EGP) did not change after autologous [from 72.9% (61.7–81.4) to 65.2% (43.3–85.2, *p* = 0.35)] or allogeneic FMT [from 70.1% (62.9–75.4) to 67.2% (43.0–84.00, *p* = 0.53)] (Supplementary Figure 2B).

We further performed correlation analysis to study the link between the FMT-induced changes in metabolic markers (weight, cholesterol levels, HOMA-IR, fasting blood glucose, and *R*_d_) and the Bray–Curtis distance, a measure of microbiome dissimilarity (from W0 to W6). We found a positive correlation between the Bray–Curtis distances and the changes in *R*_d_ in only the allogeneic FMT group (ρ = 0.622, *p* = 0.03; Supplementary Figure 3), whereas no other significant correlations were noted between variations in the metabolic markers and microbiota diversity.

Finally, we did not observe differences in the postprandial triglyceride and GLP1 levels between week 0 and week 6 in either the autologous or the allogeneic FMT group, measured during a 2-h mixed meal test (Supplementary Figures 4, 5).

#### Effect of Treatments on Fecal Bile Acids and Short-Chain Fatty Acids

To measure fecal cholesterol elimination, the cholesterol and bile acid levels were assessed in 24-h fecal samples at week −2, week 0, and week 6 (Supplementary Table 3). After 2 weeks of Mediterranean diet, we observed a substantial reduction in the 24-h fecal excretion levels of cholesterol (*p* < 0.001), dihydrocholesterol (*p* < 0.001), and coprostanol (*p* = 0.01; from W−2 to W0) (Supplementary Figure 6). However, neither form of FMT (autologous or allogeneic) induced further reductions (from W0 to W6) (Supplementary Table 3). Furthermore, no significant changes were detected in the fecal concentrations of SCFA acetate, propionate, and butyrate at any of the three time points (Supplementary Table 3).

### Impact of Mediterranean Diet and FMT Interventions on Plasma Metabolome

**FIGURE 6:**
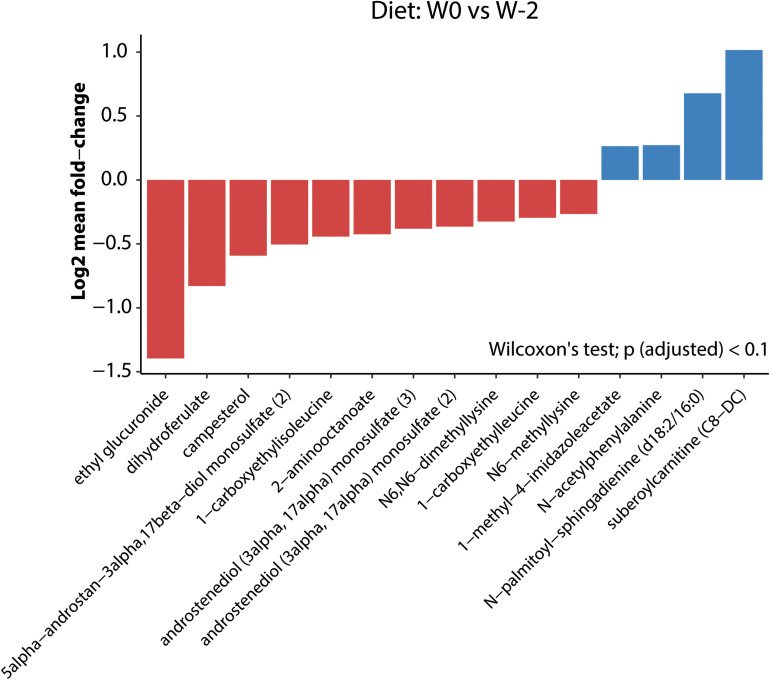
Mediterranean diet-induced changes in plasma metabolites. Log2 mean fold changes in plasma metabolites that differ significantly (*p*_adjusted_ < 0.1) between W0 (before diet) and W2 (2 weeks after initiation of the Mediterranean diet).

The Mediterranean diet induced significant changes in the levels of several fasting plasma metabolites (from W−2 to W0; Figure 6). For example, acetylphenylalanine, *N*-palmitoyl-sphingoadenine, and suberoylcarnitine were significantly increased after 2 weeks of Mediterranean diet. In contrast, ethyl glucuronide, a metabolite of ethanol, was decreased. It is not clear whether this ethanol may be of endogenous (microbial) source. Interestingly, the plant sterol campesterol, as well as derivatives of plasma amino acid metabolites lysine, leucine, and isoleucine were decreased upon Mediterranean diet. However, treatment with either autologous or allogeneic donor FMT did not differentially affect the overall plasma metabolite composition (Supplementary Figure 7). In contrast to the donor bacterial strain engraftment, we did not see that specific metabolites were transferred by the donor FMT, nor did we observe significant differences in the plasma metabolite levels at W6 between the autologous and allogeneic FMT groups (data not shown).

## Discussion

In this double-blind randomized trial, we investigated the potential synergistic effects of combining a Mediterranean diet with lean donor FMT on the gut microbiota composition, hepatic and peripheral insulin sensitivity, and plasma metabolites in treatment-naive obese subjects with metabolic syndrome. We confirm that a Mediterranean diet *per se* has a beneficial effect on metabolic parameters and results in increased relative abundances of several beneficial bacterial strains including *A. muciniphila*. The combination of this diet with lean donor FMT seemingly resulted in the engraftment of some specific FMT donor-derived bacterial species, including *Bifidobacterium* and *Bacteroides* (primary endpoint), yet failed to improve insulin sensitivity (secondary endpoint). Therefore, further studies are warranted to explore whether a longer adherence time to a Mediterranean diet or other beneficial diets (e.g., vegan or low-protein diet) together with multiple healthy donor FMTs can provide significant and durable clinical metabolic improvements to metabolic syndrome subjects.

Introduction of the Mediterranean diet was associated with alterations in specific fecal bacterial species including an increase in the levels of *A. muciniphila*. This well-studied bacterial species has previously been linked with improved glucose and cholesterol metabolism (Dao et al., 2016; Depommier et al., 2019) as well as healthy and Mediterranean diets (Liu et al., 2019; Rinott et al., 2020). Moreover, the increase in the butyrate-producing *R. hominis* corroborates other studies on Mediterranean diets (Haro et al., 2016; Ghosh et al., 2020; Meslier et al., 2020; Rinott et al., 2020) and also has been associated with beneficial effects on glucose homeostasis (Karlsson et al., 2013; Zhang et al., 2013; Meslier et al., 2020). The decrease in *C. aerofaciens* levels is in line with previous studies showing increased levels associated with DM2 and high serum cholesterol (Lahti et al., 2013; Lambeth et al., 2015) and low dietary fiber intake (Gomez-Arango et al., 2018). Upon FMT, we observed the engraftment of healthy donor-derived bacterial strains, including increased *B. pseudocatenulatum* and *G. pamelaeae* as well as *B. dorei*, which are all linked to anti-inflammatory and beneficial metabolic effects (Moya-Pérez et al., 2015; Selma et al., 2017; Yoshida et al., 2018; Sanchis-Chordà et al., 2019). In contrast, following donor FMT, decreased levels of the opportunistic pathogen *D. piger* often seen in DM2 were observed in recipients (Qin et al., 2012; Doumatey et al., 2020). Despite the increased fiber intake, the Mediterranean diet did not result in increased fecal SCFA levels in this study. Although a Mediterranean diet has previously been associated with higher fecal SCFA levels (De Filippis et al., 2015), others corroborate our findings (Wu et al., 2016; Meslier et al., 2020). Wu et al. (2016) proposed the concept of a restrictive microbiota associated with westernized societies as an explanation for the lack of increased SCFA formation despite additional substrate intake. Moreover, the unchanged fecal SCFA levels could be caused by an increased utilization or rapid absorption by the colonocytes (Sakata, 2019).

Our group previously demonstrated an increase in peripheral insulin sensitivity (*R*_d_) upon lean donor FMT (Vrieze et al., 2012; Kootte et al., 2017) while subjects were adhering to their habitual (omnivorous) diet. To our surprise, we observed no synergistic effect of combining Mediterranean diet with lean donor FMT on metabolic parameters including peripheral and hepatic insulin sensitivity, although a large standard deviation (Supplementary Figure 2) was observed, suggesting responders and non-responders upon this combined intervention. However, the sample size precluded further *post hoc* analyses. Previous data have demonstrated that the effect of a FMT is controlled by the baseline microbiota composition (Li et al., 2016; Kootte et al., 2017) and that subjects with a low baseline diversity have greater metabolic improvement upon interventions (Cotillard et al., 2013; Kootte et al., 2017; Yu et al., 2020). Despite the short-period of dietary intervention (as compared to other studies, e.g., those of Meslier et al. (2020) with 8 weeks of Mediterranean diet intervention and Ghosh et al., 2020 with 1 year), the Mediterranean diet intake provoked clear changes in the gut microbiota composition, whereas the introduction of a healthy donor microbiota resulted in modest changes in the microbiome and did not significantly increase the microbiome species diversity.

Thus, we postulate that the diet-induced changes in the gut microbiota (enrichment in the beneficial commensal species *Bacteroides*, *A. muciniphila*, and *R. hominis*) and metabolic profiles (decrease in fasting glucose, cholesterol, body weight, and a trend toward lower HOMA-IR index) render the host less susceptible to FMT-induced changes and therefore attenuates the (previously observed) beneficial effect of a lean donor FMT on both hepatic and peripheral insulin sensitivity. We hypothesize that the gut microbial system is “locked” after the introduction of a beneficial Mediterranean diet, precluding additional beneficial metabolic effects of donor FMT. Indeed, a higher biodiversity in an ecosystem is associated with increased resilience, thus higher tolerance to environmental perturbations (in this case represented by engraftment of exogenous bacterial species) (Mosca et al., 2016). This hypothesis is underscored by the fact that only a few donor strains were able to engraft in the gut as compared to other studies without dietary interventions prior to FMT (Li et al., 2016). Finally, we observed a significant positive correlation between the changes (between W0 and W6) in the Bray–Curtis distance, a measure of microbiome dissimilarity, and insulin sensitivity (*R*_d_ changes) solely after lean donor FMT and not in the autologous FMT group. This indicates that, when the microbiota composition/diversity still exhibits room for change, we do observe greater alterations in insulin sensitivity after lean donor FMT.

Moreover, we cannot exclude that the difference in the effects of allogeneic or autologous FMT is masked by the introduction of a “less diabetogenic” microbiota in the autologous FMT group after 2 weeks on Mediterranean diet. In line with this, Rinott et al. (2020) reported that autologous FMT utilizing fecal samples collected at the end of a 6-month Mediterranean diet intervention ameliorated the metabolic profiles of obese dyslipidemic patients until 8 months afterward. Of note is that the microbiome intervention in the present study consisted of a single fresh (non-processed) duodenal lean donor FMT, in line with previous studies from our group (Vrieze et al., 2012; Kootte et al., 2017). More recent microbiota intervention studies in obese populations, however, investigated the effect of oral encapsulated (processed) feces, repeated transplantations, and the usage of multiple donors (Allegretti et al., 2020; Leong et al., 2020; Yu et al., 2020). Although the metabolic effects in these studies were variable, the microbial shifts might be more pronounced and durable. Perhaps, in the present study, a more robust microbiota intervention (e.g., multiple FMTs or lean donor FMTs prior to the dietary intervention) could have resulted in a more distinct effect of the lean donor microbiota on top of the rigorous change in diet.

It is known that different levels of plasma metabolites corroborate with the changes in food intake. Hereof, the plasma levels of lysine, leucine, and isoleucine derivatives (being the amino acids mainly found in meat and cheese) were significantly lowered after 2 weeks of Mediterranean diet. In line with this, previous studies also described a decrease in branched-chain amino acids after a Mediterranean or a vegan diet (Draper et al., 2018; Meslier et al., 2020).

Similarly to what we observed for the metabolic parameters after FMT, no additive effects of FMT on plasma metabolome were detected, indicating that the Mediterranean diet-induced changes in the metabolic profiles overrides the effect of donor bacterial species engraftment. The observed decrease in fecal plant sterol levels (most specifically campesterol) suggests a change in intestinal cholesterol absorption, which was surprising in view of the major decrease in total sterol excretion. Although the Mediterranean diet is low in cholesterol, the observed decrease in the total sterol output was more than 1,000 mg/day (Supplementary Figure 4). As a normal cholesterol intake amounts to around 300 mg/day, this changed intake cannot account for the effect, and we therefore hypothesize that cholesterol synthesis itself must have decreased, accounting for the decrease in the plasma levels of total cholesterol, LDL, and HDL. In contrast to the recent study of Meslier et al. (2020), we did not observe changes in the fecal bile acid output. Perhaps a 2-week exposure to the Mediterranean diet was not enough to translate the decreased cholesterol synthesis to a decrease in bile acid synthesis.

Our study has several limitations. Firstly, a notable difference between our current and previous FMT studies is the insulin sensitivity prior to the administration of FMT (Vrieze et al., 2012; Kootte et al., 2017). Although these former studies had comparable inclusion criteria and the metabolic profiles at baseline were largely comparable, the median *R*_d_ after 2 weeks of Mediterranean diet just before applying donor FMT (week 0) was much higher in the current study (25.8 and 26.2 vs. 41.0 μmol kg^–1^ min^–1^), although still in the range found in obese individuals (Vrieze et al., 2012; Ter Horst et al., 2015). In line with the significant increase in HOMA-IR between week −2 and week 0, we can speculate that this higher *R*_d_ results from a 2-week Mediterranean diet consumption. Nonetheless, ideally, we should have determined insulin sensitivity at W−2 as well (thus, before starting the Mediterranean diet) in order to test this hypothesis; however, due to ethical restrictions, we were only allowed to perform the hyperinsulinemic–euglycemic clamps twice. Secondly, in line with our previous studies (Vrieze et al., 2012; Kootte et al., 2017), we included Caucasian male subjects only. Although this was done to minimize the well-known effects of ethnicity and sex (hormones) on the gut microbiota composition (Deschasaux et al., 2018; Yoon and Kim, 2021), this may have reduced generalization to the general population. Thirdly, although the subjects were monitored by a hospital dietitian for dietary adherence, data on the actual dietary intake were retrieved from self-reported online nutritional diaries. Prior studies have demonstrated that self-administered dietary records may underestimate the energy intake in overweight and obese individuals (Lichtman et al., 1990; Schoeller, 1995; Bedard et al., 2004; Freedman et al., 2014). However, as all dietary records were checked by the dietitian on the same week, abnormalities were discussed immediately with the subject and corrected if necessary. Finally, despite the fact that no total calorie restriction was promoted, we observed a decrease in total energy intake in the first 2 weeks. Although the total amount of weight loss in these weeks was not that pronounced and other studies corroborate the beneficial metabolic health effects of the Mediterranean diet (including non-calorie-restricted diets) (Salas-Salvadó et al., 2011; Estruch et al., 2018), the decreased total energy intake *per se* may have influenced the metabolic outcomes.

## Conclusion

We confirm previous studies showing a beneficial effect of a Mediterranean diet on metabolic markers and gut microbiota composition in metabolic syndrome subjects. Although we observed (donor FMT-induced) engraftment of some bacterial species in the feces on top of this diet, we did not observe synergistic beneficial metabolic effects in this small randomized controlled trial. Therefore, these data warrant further study by combining specific diets with more frequently administered and/or different microbiome interventions (like repeated FMTs, specific bacterial species, or donor selection) to optimize microbiota changes and potential metabolic effects. Alternatively, given the previous indications that a low baseline microbiota diversity results in a greater metabolic response, pretreatment with antibiotics before microbial intervention is still an interesting, although challenging, concept.

## Data Availability Statement

The data have been deposited at the European Nucleotide Archive (ENA) under the accession number PRJEB44237.

## Ethics Statement

The studies involving human participants were reviewed and approved by METC AMC (Amsterdam University Medical Center, Amsterdam, Netherlands). The patients/participants provided their written informed consent to participate in this study.

## Author Contributions

AK, AG, and MN designed the study. AK, IA, JW, WF, ER, JH, SM, MK, JL, AS, HH, TS, LD, BH, JH, and PO’T performed the research. AK and EA performed the statistical analysis. AK, AG, and MN drafted the manuscript. All authors critically reviewed the manuscript.

## Conflict of Interest

MN is in the Scientific Advisory Board of Caelus Pharmaceuticals, Netherlands and Kaleido, United States. None of these are directly relevant to the current paper. The remaining authors declare that the research was conducted in the absence of any commercial or financial relationships that could be construed as a potential conflict of interest.

## Footnotes

1 https://mijn.voedingscentrum.nl/nl/eetmeter/

2 https://doi.org/10.2144/04365ST04

3 https://github.com/biobakery/kneaddata

## Supplementary Material

Supplementary Figure 1 Subcutaneous tissue inflammation. Change in gene expression in subcutaneous adipose tissue between week 0 and week 6 for both the autologous and allogeneic FMT group. Each line represents an individual subject. None of the genes in our panel changed significantly (rest of the genes not shown). CCL2, chemokine-ligand 2; IL-10, interleukin-10; IRS1, insulin receptor substrate 1; CD11, cluster of Differentiation 11, TNF, tumor necrosis factor; IL-6, interleukin-6, CD68, cluster of differentiation 68.

Supplementary Figure 2 Peripheral and hepatic insulin sensitivity. This figure shows changes in insulin sensitivity between weeks 0 and 6 for both the autologous and allogeneic treatment group. In both groups there were no statistically significant changes in peripheral (*R*_d_) or hepatic (endogenous glucose production –EGP- suppression) insulin sensitivity. There were also no differences between the autologous and allogeneic FMT group. Box and whisker, 10–90th percentile.

Supplementary Figure 3 Correlation change in *R*_d_ and Bray–Curtis distance. This figure depicts the positive correlation between Bray Curtis distance (a measure of microbiome dissimilarity) and change in *R*_d_ (a measure of peripheral insulin sensitivity) in only the allogeneic FMT group (Rho 0.622, *p* = 0.03).

Supplementary Figure 4 Postprandial triglycerides. This figure shows triglyceride excursions after mixed meal for week 0 and week 6 for both autologous and allogeneic FMT group (line plot, median + IQR). The box and whisker plots (min–max) on the right represent area under curves (AUC) for both groups at the different time points. There were no significant differences between the two groups and within the groups at any time point.

Supplementary Figure 5 Postprandial GLP-1. This figure shows GLP-1 excursions after mixed meal for week 0 and week 6 for both autologous and allogeneic FMT group (line plot, median + IQR). The box and whisker plots (min–max) on the right represent area under curves (AUC) for both groups at the different time points. There were no significant differences between the two groups and within the groups at any time point.

Supplementary Figure 6 24 h fecal sterol excretion. This figure shows 24-h fecal cholesterol, coprostanol and DiH-cholesterol excretion in the first 2 weeks of the Mediterranean diet (whole group). Box and whisker plots, min–max.

Supplementary Figure 7 Fecal microbiota transplantation (FMT) induced changes in plasma metabolome. Effect of treatment with either autologous **(A)** or allogeneic donor **(B)** FMT on overall plasma metabolite composition.

Supplementary Table 1 Primers used to measure the relative expression of *IL-10, CCL2, CD68, CD11c, IRS1, TNF*, and *IL-6*.

Supplementary Table 2 Macronutrient intake before-, during- and after the Mediterranean diet, assessed using self-reported only nutritional diaries. Diet-induced changes in energy, fat, saturated fat, carbohydrate and fiber intake did not significantly differ between the two FMT groups.

Supplementary Table 3 Fecal bile acid, -cholesterol and -short chain fatty acid concentrations, expressed as medians and interquartile ranges. Effect of Mediterranean diet only (W−2 to W0, whole group) and with addition of FMT (W0 to W6, split to autologous FMT and allogeneic FMT). There were no statistically significant differences in any of the above parameters between the autologous and allogeneic group at any time point.
