# Supplementary figures and images for: Effect of Fecal Microbiota Transplantation Combined With Mediterranean Diet on Insulin Sensitivity in Subjects With Metabolic Syndrome
Source: Front Microbiol. 2021 Jun 10;12:662159. doi: 10.3389/fmicb.2021.662159 (PMC8222733; doi:10.3389/fmicb.2021.662159)

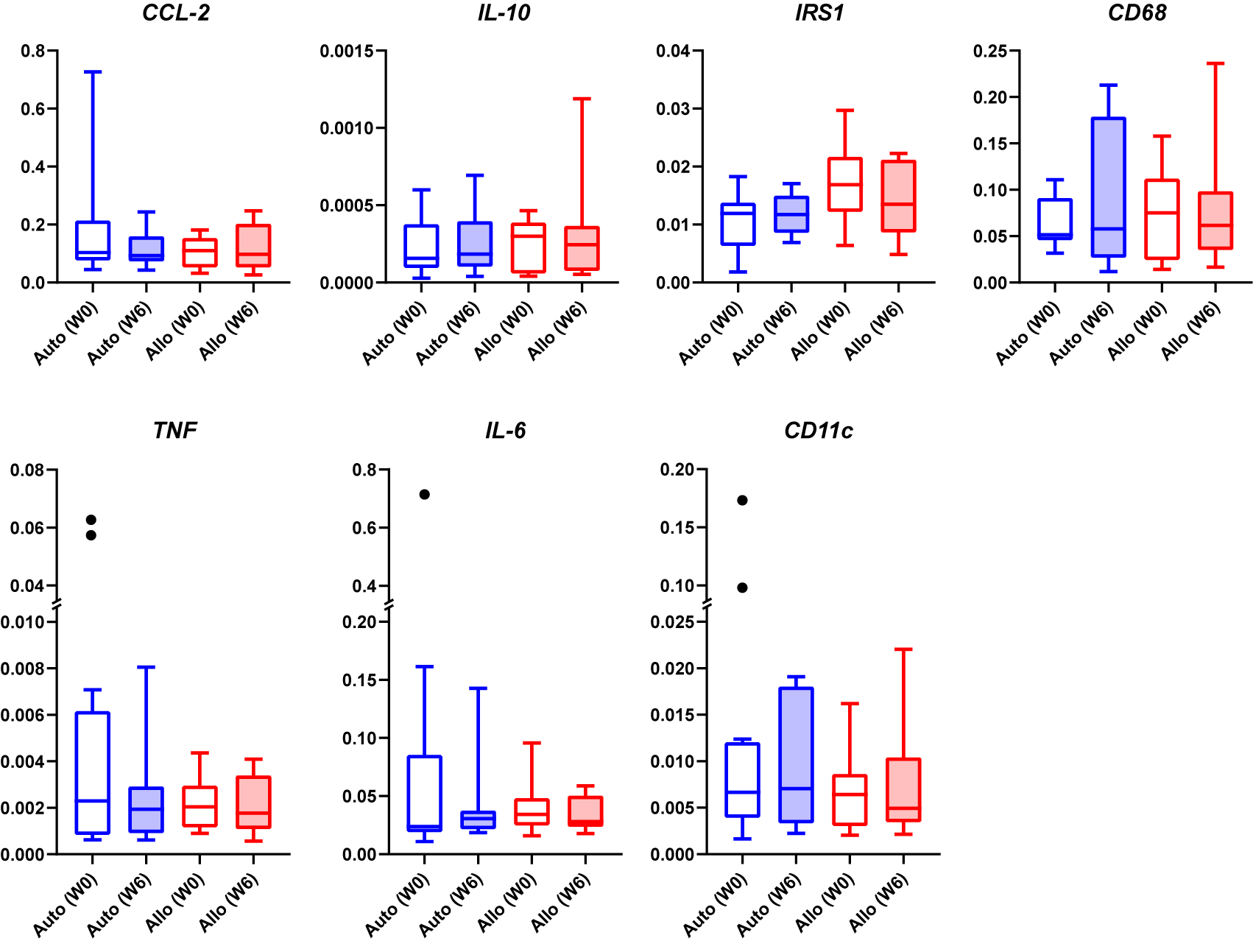

Supplement: Supplementary Figure 1 — Subcutaneous tissue inflammation. Change in gene expression in subcutaneous adipose tissue between week 0 and week 6 for both the autologous and allogeneic FMT group. Each line represents an individual subject. None of the genes in our panel changed significantly (rest of the genes not shown). CCL2, chemokine-ligand 2; IL-10, interleukin-10; IRS1, insulin receptor substrate 1; CD11, cluster of Differentiation 11, TNF, tumor necrosis factor; IL-6, interleukin-6, CD68, cluster of differentiation 68. [file Image_1.TIF]

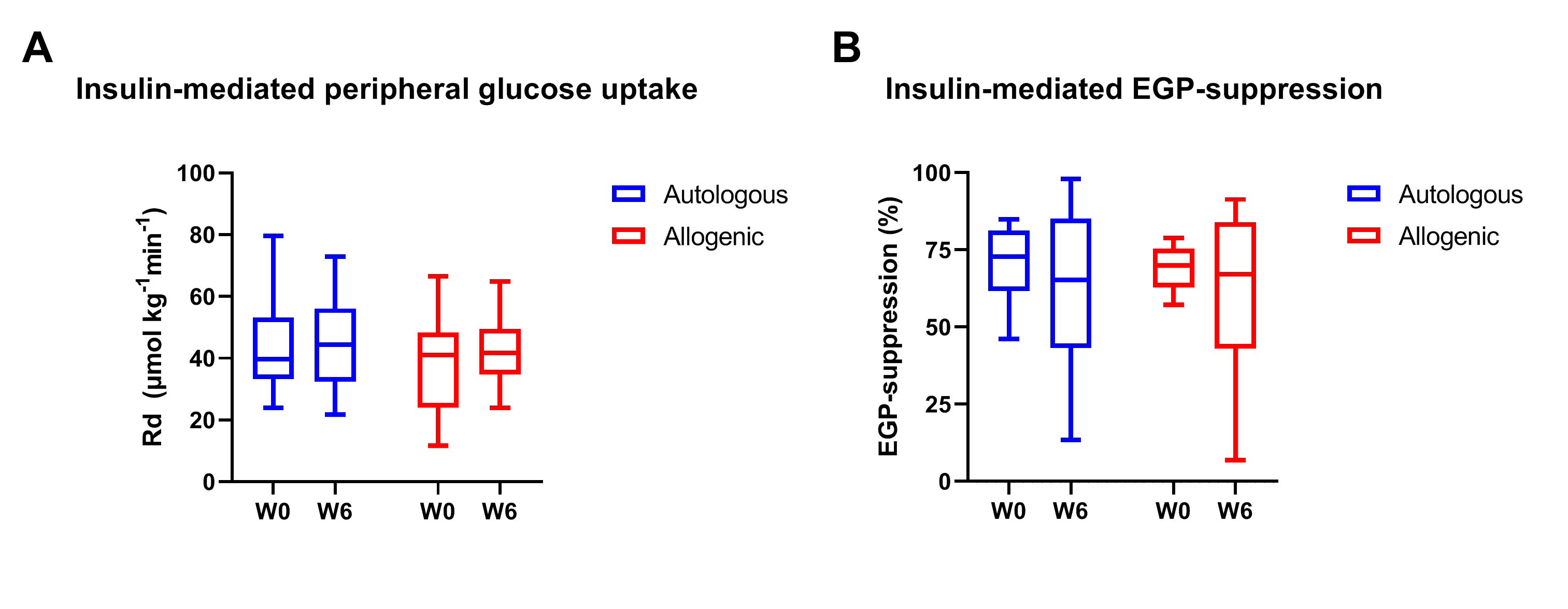

Supplement: Supplementary Figure 2 — Peripheral and hepatic insulin sensitivity. This figure shows changes in insulin sensitivity between weeks 0 and 6 for both the autologous and allogeneic treatment group. In both groups there were no statistically significant changes in peripheral (Rd) or hepatic (endogenous glucose production –EGP- suppression) insulin sensitivity. There were also no differences between the autologous and allogeneic FMT group. Box and whisker, 10–90th percentile. [file Image_2.TIF]

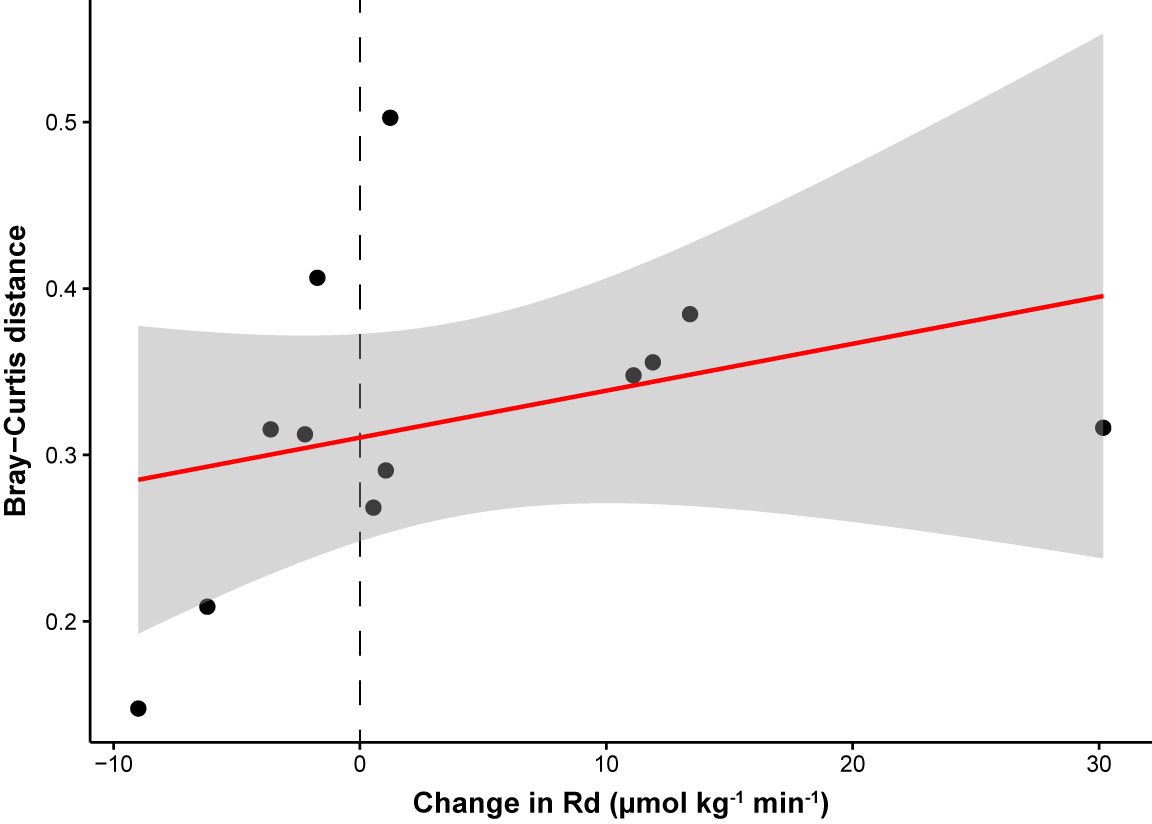

Supplement: Supplementary Figure 3 — Correlation change in Rd and Bray–Curtis distance. This figure depicts the positive correlation between Bray Curtis distance (a measure of microbiome dissimilarity) and change in Rd (a measure of peripheral insulin sensitivity) in only the allogeneic FMT group (Rho 0.622, p = 0.03). [file Image_3.TIF]

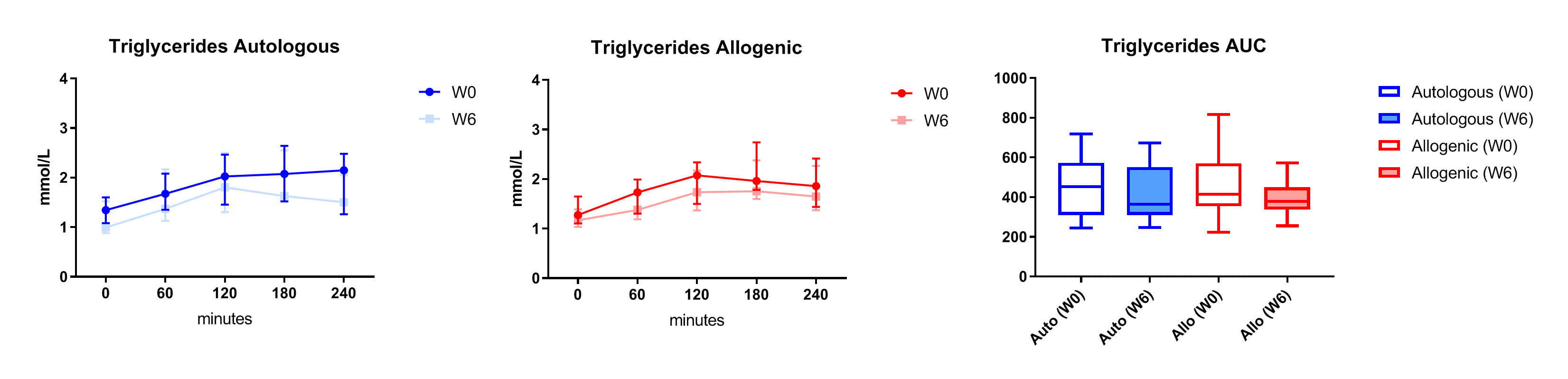

Supplement: Supplementary Figure 4 — Postprandial triglycerides. This figure shows triglyceride excursions after mixed meal for week 0 and week 6 for both autologous and allogeneic FMT group (line plot, median + IQR). The box and whisker plots (min–max) on the right represent area under curves (AUC) for both groups at the different time points. There were no significant differences between the two groups and within the groups at any time point. [file Image_4.TIF]

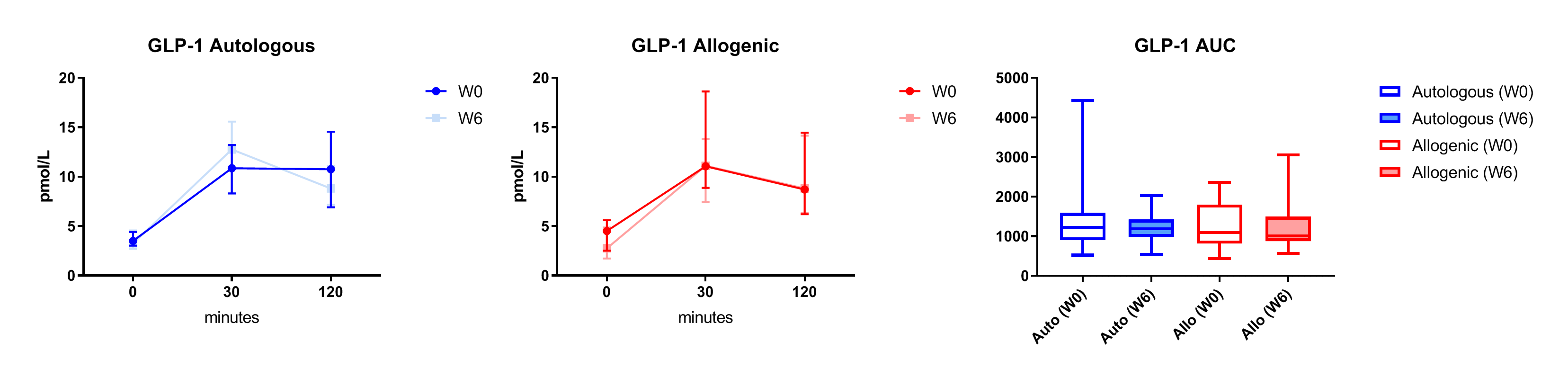

Supplement: Supplementary Figure 5 — Postprandial GLP-1. This figure shows GLP-1 excursions after mixed meal for week 0 and week 6 for both autologous and allogeneic FMT group (line plot, median + IQR). The box and whisker plots (min–max) on the right represent area under curves (AUC) for both groups at the different time points. There were no significant differences between the two groups and within the groups at any time point. [file Image_5.TIF]

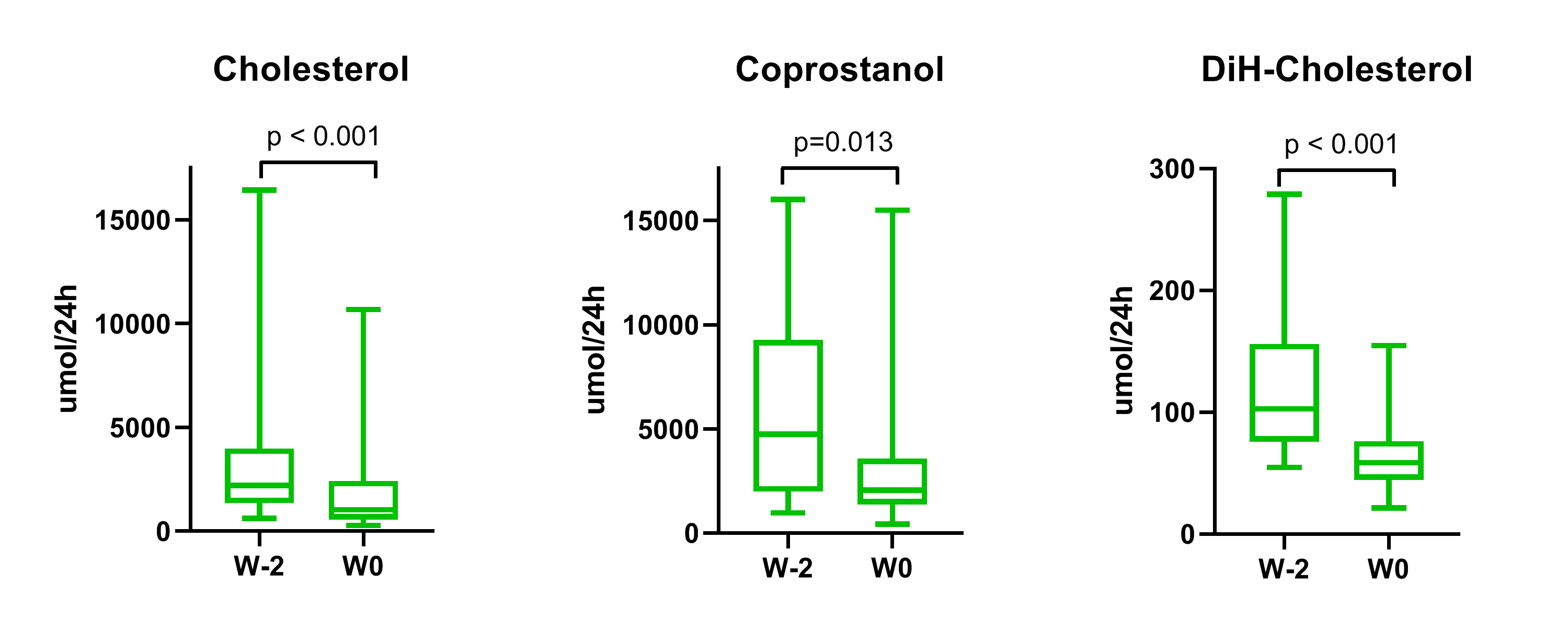

Supplement: Supplementary Figure 6 — 24 h fecal sterol excretion. This figure shows 24-h fecal cholesterol, coprostanol and DiH-cholesterol excretion in the first 2 weeks of the Mediterranean diet (whole group). Box and whisker plots, min–max. [file Image_6.TIF]

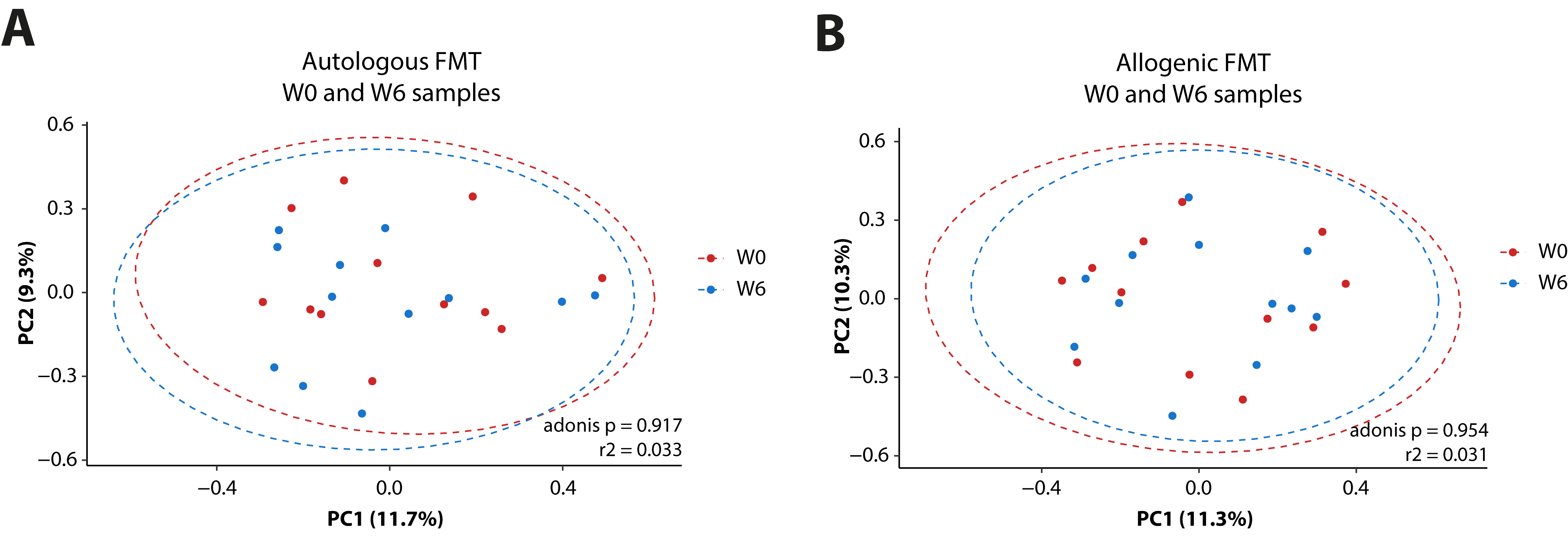

Supplement: Supplementary Figure 7 — Fecal microbiota transplantation (FMT) induced changes in plasma metabolome. Effect of treatment with either autologous (A) or allogeneic donor (B) FMT on overall plasma metabolite composition. [file Image_7.TIF]
