# Supplementary material for: Effect of Fecal Microbiota Transplantation Combined With Mediterranean Diet on Insulin Sensitivity in Subjects With Metabolic Syndrome
Source: Front Microbiol. 2021 Jun 10;12:662159. doi: 10.3389/fmicb.2021.662159 (PMC8222733; doi:10.3389/fmicb.2021.662159)
Supplement: Supplementary Table 1 — Primers used to measure the relative expression of IL-10, CCL2, CD68, CD11c, IRS1, TNF, and IL-6. [file Table_1.docx]

**SUPPLEMENTAL TABLES**

| **Primers** |  |
| --- | --- |
| 36B4 Forward | ACGGGTACAAACGAGTCCTG |
| 35B4 Reverse | GCCTTGACCTTTTCAGCAAG |
| CD68 Forward | CCC CAA CAA AAC CAA GGT CC |
| CD68 Reverse | GGA GGT CCT GCA TGA ATC CAA A |
| IL-6 Forward | CTG CAG AAA AAG GCA AAG AAT CTA |
| IL-6 Reverse | GTT GTC ATG TCC TGC AGC C |
| CCL2 Forward | TGTCCCAAAGAAGCTGTGATC |
| CCL2 Reverse | ATTCTTGGGTTGTGGAGTGAG |
| IL-10 Forward | GAT GCC TTC AGC AGA GTG AA |
| IL-10 Reverse | GCA ACC CAG GTA ACC CTT AAA |
| IRS1 Forward | TAT GCC AGC ATC AGT TTC CA |
| IRS1 Reverse | TTT GCT GAG GTC ATT TAG GTC TT |
| TNF Forward | CAGCCTCTTCTCCTTCCTGAT |
| TNF Reverse | GCCAGAGGGCTGATTAGAGA |
| CD11c Forward | TGA GCA GTG AGA ACA ACA CTC C |
| CD11c Reverse | AGC ATA CTT CAC CGG GAG C |

**Supplemental table 1:** Primers used to measure the relative expression of *IL-10, CCL2, CD68, CD11c, IRS1, TNF* and *IL-6*.

|  | **Pre diet** | **Diet** | **Post diet** | **P  pre \| diet** | **P  diet \| post** | **P  pre \| post** |
| --- | --- | --- | --- | --- | --- | --- |
| **Energy (kcal)** | 2193 [1734 – 2644] | 1820 [1602 – 2009] | 1987 [1683 – 2214] | **0.01** | **0.01** | 0.17 |
| **Fat total (gram)** | 85 [68 -109] | 81 [66 – 100] | 75 [71 -93] | 0.66 | 0.87 | 0.16 |
| **Fat saturated (gram)** | 28 [19 -39] | 20 [17 – 23] | 25 [20 -28] | **0.01** | **0.01** | 0.06 |
| **Carbohydrates (gram)** | 216 [167 – 272] | 158 [148 – 191] | 180 [166 – 228] | **0.01** | **0.01** | 0.24 |
| **Fibers (gram)** | 20 [17 – 25] | 24 [23 – 28] | 23 [17 – 27] | **0.02** | 0.18 | 0.26 |
| **Proteins (gram)** | 90 [73 – 115] | 81 [75 – 94] | 88 [72 – 111] | 0.123 | 0.17 | 0.28 |

**Supplemental table 2**: Macronutrient intake before-, during- and after the Mediterranean diet, assessed using self-reported only nutritional diaries. Diet-induced changes in energy, fat, saturated fat, carbohydrate and fiber intake did not significantly differ between the two FMT groups.

|  | **Diet only** | | | **Diet + autologous FMT** | | | **Diet + allogenic FMT** | | |
| --- | --- | --- | --- | --- | --- | --- | --- | --- | --- |
|  | W-2 (n=24) | W0 (n= 24) | p | W0 (n=12) | W6 (n=12) | p | W0 (n=12) | W6 (n=12) | p |
| **Coprostanol (umol/24h)** | 4752 [1990 -9263] | 2062 [1382 – 3564] | **0.01** | 1692 [1073 -2916] | 1390 [708 – 3093] | 0.21 | 2777 [1541 – 4508] | 2333 [1728 – 3282] | 0.81 |
| **Cholesterol (umol/24h)** | 2202 [1357 – 3983] | 1025 [562 – 2433] | **<0.001** | 1004 [495 -2434] | 788 [561 -1992] | 0.70 | 1026 [598 – 2482] | 735 [474 – 2674] | 0.16 |
| **DiH-cholesterol (umol/24h)** | 103 [76 – 156] | 59 [44 – 76] | **<0.001** | 55 [40 – 70] | 48 [27 – 78] | 0.39 | 65 [48 – 83] | 75 [45 – 101] | 0.43 |
| **Cholate (umol/24h)** | 34 [26 -94] | 24 [12 – 52] | 0.58 | 39 [15 – 70] | 23 [11 – 31] | 0.35 | 19 [ 12 – 28] | 14 [11 – 31] | 0.40 |
| **Chenodexycholate (umol/24h)** | 49 [25 – 99] | 62 [33- 102] | 0.96 | 73 [40 0 131] | 38 [25 – 66] | 0.12 | 38 [27 -100] | 39 [20 -66] | 0.21 |
| **Iso-lithocholate (umol/24h)** | 227 [164 – 379] | 226 [166 – 314] | 0.75 | 218 [171 – 297] | 185 [144 –  329] | 0.39 | 252 [160 – 346] | 244 [152 – 349] | 0.70 |
| **Lithocholate (umol/24h)** | 950 [749-1574] | 858 [598 – 1273] | 0.61 | 1036 [570 – 1472] | 638 [549 – 1021] | 0.24 | 732 [601 – 1182] | 1014 [529 – 1427] | 1.00 |
| **Deoxycholate (umol/24h)** | 1304 [860- 2921] | 1233 [847 – 1940] | 0.32 | 1413 [802 – 2149] | 818 [550 – 128] | 0.24 | 1126 [881 – 1561] | 1142 [561 – 1661] | 0.08 |
| **Butyrate (umol/g wet weight corr)** | 21 [14 – 29] | 18 [10 – 29] | 0.56 | 18 [10 – 30] | 23 [6 – 28] | 1.00 | 17 [9 – 25] | 15 [8 – 21] | 0.79 |
| **Acetate (umol/g wet weight corr)** | 88 [78 – 103] | 74 [55 – 96] | 0.25 | 74 [51 – 127] | 85 [72 – 106] | 0.93 | 73 [56 – 89] | 76 [58 – 99] | 0.93 |
| **Propionate(umol/g wet weight corr)** | 29 [20 – 42] | 20 [11 – 34] | 0.17 | 23 [11 – 36] | 28 [17 – 37] | 0.59 | 19 [11 – 25] | 24 [13 – 32] | 0.93 |

**Supplemental table 3**: Fecal bile acid, -cholesterol and -short chain fatty acid concentrations, expressed as medians and interquartile ranges. Effect of Mediterranean diet only (W-2 to W0, total group) and with addition of FMT (W0 to W6, split to autologous FMT and allogenic FMT). There were no statistically significant differences in any of the above parameters between the autologous and allogenic group at any time point
