# Supplementary material for: Effect of Fecal Microbiota Transplantation Combined With Mediterranean Diet on Insulin Sensitivity in Subjects With Metabolic Syndrome
Source: Front Microbiol. 2021 Jun 10;12:662159. doi: 10.3389/fmicb.2021.662159 (PMC8222733; doi:10.3389/fmicb.2021.662159)
Supplement: Supplementary file 9 [file Data_Sheet_1.docx]

**SUPPLEMENTAL DATA**

**Supplemental methods: Mediterranean diet**

The Mediterranean diet guidelines used in this study were based on the PREDIMED study*

The general guidelines to follow the Mediterranean diet that dietitians provided to

participants and that were used to compose the food boxes included the following positive recommendations:

a) abundant use of olive oil for cooking and dressing dishes;

b) consumption of ≥ 2 daily servings of vegetables (at least one of them as fresh vegetables in a salad)

c) ≥ 2-3 daily servings of fresh fruits (including natural juices);

d) 1-3 weekly servings of legumes;

e) ≥ 3 weekly servings of fish or seafood (at least one serving of fatty fish)

f) ≥ 1 portion nuts (raw and unsalted) /day; g) select white meats (poultry without skin or rabbit) instead of red meats or processed meats (burgers, sausages)

Negative recommendations are also given to eliminate or limit the consumption of cream, butter, margarine, cold meat, pate, duck, carbonated and/or sugared beverages, pastries, industrial bakery products (such as cakes, donuts, or cookies), industrial desserts (puddings, custard), French fries or potato chips, and out-of-home pre-cooked cakes and sweets

*Ad libitum* consumption was allowed for the following food items: nuts (raw and unsalted), eggs, fish (recommended for daily intake), seafood, low-fat cheese, chocolate (only black chocolate, with more than 50% cocoa), and whole-grain cereals. Limited consumption (≤1 serving per week) was advised for cured ham, red meat (after removing all visible fat), and cured or fatty cheeses.

There was no specific energy restriction.

*Estruch R, Ros E, Salas-Salvadó J, et al. Primary Prevention of Cardiovascular Disease with a Mediterranean Diet Supplemented with Extra-Virgin Olive Oil or Nuts. *N Engl J Med*. 2018;378(25):e34. doi:10.1056/NEJMoa1800389
